# Serial serum calcium dynamics predict delayed hydrocephalus after spontaneous subarachnoid hemorrhage: development and validation of a clinical nomogram in an observational cohort

**DOI:** 10.3389/fneur.2026.1762189

**Published:** 2026-03-24

**Authors:** Di Wu, Xingye Zhai, Xinyue Zhang, Xu Han, Lingyu Hao, Cong Wang, Sihan Wang, Zhen Wang, Zengguang Wang, Yi Wang

**Affiliations:** 1 Department of Neurosurgery, Tianjin Medical University General Hospital, Tianjin, China; 2 Department of Neurosurgery, Tianjin Huanhu Hospital, Tianjin, China; 3 Key Laboratory of Post-trauma Neuro-repair and Regeneration in Central Nervous System, Ministry of Education, Key Laboratory of Injuries, Variations and Regeneration of Nervous System, Tianjin Neurological Institute, Tianjin, China; 4 Department of Neurosurgery, North China University of Science and Technology Affiliated Hospital, Tangshan, China; 5 Department of Neurosurgery, Qinghai University Affiliated Hospital, Xining, Qinghai, China

**Keywords:** biomarker, delayed hydrocephalus, nomogram, prediction model, serum calcium, subarachnoid hemorrhage

## Abstract

**Background:**

Delayed hydrocephalus, a severe complication following spontaneous subarachnoid hemorrhage (SAH), lacks reliable early biomarkers. Calcium dysregulation has emerged as a potential mediator of secondary brain injury, but the predictive value of serum calcium dynamics for delayed hydrocephalus remains unexplored.

**Methods:**

In this observational cohort study, we analyzed serial serum calcium measurements (admission, 72 h, and 7 days post-ictus) in SAH patients monitored for 90 days to identify delayed hydrocephalus, defined by radiological ventricular enlargement and clinical neurological deterioration requiring cerebrospinal fluid diversion. Predictive accuracy was evaluated using ROC analysis, and a clinical decision pathway integrating calcium thresholds with neurological assessments was proposed. An admission-variable nomogram was developed using LASSO and logistic regression modeling for early risk stratification. The model was externally validated in a cohort from two hospitals.

**Results:**

Among 302 patients analyzed, delayed hydrocephalus occurred in 24.2%. Serum calcium levels declined over the first week after SAH and were consistently lower at all measured time points among patients who developed delayed hydrocephalus (*p* < 0.001). Serum calcium at 72 h showed the highest predictive accuracy (AUC = 0.854), superior to conventional severity scales, with a cutoff ≤7.65 mg/dL (sensitivity 85.6%, specificity 80.3%). The admission-based nomogram incorporating admission serum calcium, hypertension, Hunt-Hess grade, smoking history, dyslipidemia, alcohol use and diabetes demonstrated excellent performance with an AUC > 0.93 in internal validation and an AUC of 0.898 in external validation.

**Conclusion:**

Dynamic serum calcium levels, particularly hypocalcemia at 72 h, were strongly associated with delayed hydrocephalus after spontaneous SAH and showed good discriminatory performance. An admission-based nomogram provides early individualized risk stratification. These accessible tools could support clinical decision-making, but the proposed calcium cutoff may vary across laboratory assays and therefore warrants multicenter and cross-platform validation (with local calibration where appropriate) and further mechanistic studies.

## Introduction

Spontaneous subarachnoid hemorrhage (SAH), a life-threatening cerebrovascular emergency caused by ruptured aneurysms or vascular malformations, triggers complex pathophysiological cascades when blood extravasates into the subarachnoid space (1). Despite advances in acute management, delayed hydrocephalus, a debilitating complication that affects 20%–30% of SAH survivors, remains a major contributor to long-term disability and mortality (2). Delayed hydrocephalus typically manifests weeks to months after the ictus and is characterized by impaired cerebrospinal fluid (CSF) absorption (3–5). This is caused by dysfunction of arachnoid granulations, ventricular obstruction, or fibrotic changes induced by hemoglobin degradation products (3, 6, 7). The insidious progression of delayed hydrocephalus frequently leads to diagnostic delays until permanent neurological damage is established, highlighting the imperative for reliable, accessible biomarkers capable of risk stratification during the acute phase (8, 9).

Emerging evidence suggests that calcium dysregulation may contribute to in mediating secondary brain injury following SAH. As a ubiquitous second messenger, intracellular calcium regulates cerebral vascular tone, neuronal excitability, and apoptotic pathways (10, 11). Although intracellular Ca^2+^ signaling and circulating (serum) calcium are physiologically distinct, we focus on serum calcium in this study because it reflects systemic calcium homeostasis and stress-related endocrine/inflammatory responses, and may serve as an accessible surrogate marker of the overall pathophysiological milieu after SAH. SAH-induced vasoactive substances, such as endothelin-1 and serotonin, trigger pathological calcium influx through voltage-gated and receptor-operated channels, leading to intracellular calcium overload (12, 13). Such calcium-related signaling has been associated with cerebral vasospasm, disrupts blood–brain barrier (BBB) integrity, and promotes neuroinflammation—processes implicated in delayed hydrocephalus development (14, 15). Notably, hypocalcemia at admission has been found to correlate with hematoma expansion in intracerebral hemorrhage and is associated with poorer functional outcomes (16). However, the temporal trajectory of serum calcium fluctuations post-SAH and its specific association with delayed hydrocephalus remain unexplored, representing a critical knowledge gap.

Current methods for predicting delayed hydrocephalus rely on invasive CSF dynamics assessment or delayed neuroimaging findings, both of which are impractical for real-time clinical decision-making (17, 18). Although inflammatory markers such as IL-6 and MMP-9 have prognostic potential, their measurement requires specialized assays that are seldom available in routine practice (19, 20). In contrast, serum calcium measurement is a rapid, cost-effective, and widely accessible alternative that can be monitored serially in clinical practice. Preliminary studies suggest that dynamic serum calcium changes may reflect evolving neurovascular injury; however, no systematic investigation has yet evaluated their predictive utility for delayed hydrocephalus (16, 21). In addition, a nomogram is a simple graphical representation of a multivariable prediction model that provides an individualized probability of an outcome at the bedside (22). Given that delayed hydrocephalus often develops weeks after SAH and that the most informative calcium measurement may only become available after 72 h, an admission-based nomogram using routinely collected baseline variables could support early risk stratification and facilitate clinical implementation of our findings.

This pre-specified observational cohort study investigates the association between serum calcium dynamic changes and the development of delayed hydrocephalus in patients with SAH, and further develops an admission-based nomogram for early individualized risk stratification. We first characterize longitudinal serum calcium patterns during the acute phase (Days 1–7 post-ictus) and their correlation with neurological severity assessed through Glasgow Coma Scale (GCS) and Hunt-Hess grading. Furthermore, we evaluate whether dynamic changes in serum calcium serve as predictive biomarkers for delayed hydrocephalus diagnosis within 90 days, as confirmed by standardized neuroimaging criteria. These findings could provide critical insights into the calcium-mediated mechanisms underlying hydrocephalus formation following SAH.

## Methods

### Study design and participants

**Figure 1:**
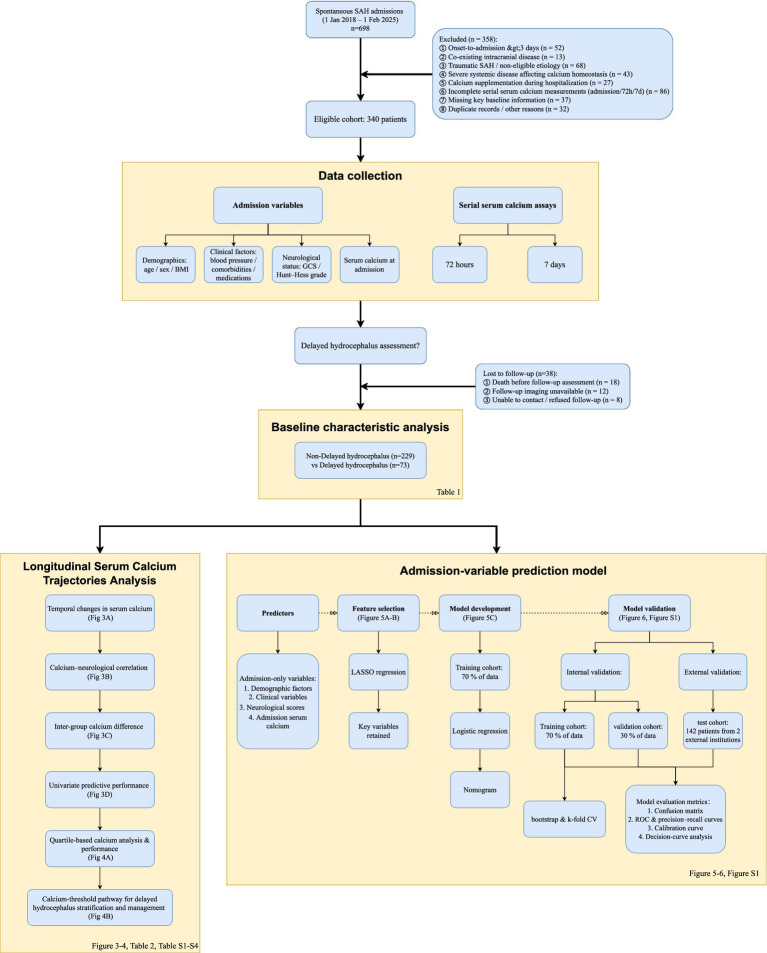
Study flow diagram. This figure illustrates the full methodological framework of the study, from cohort enrollment and data acquisition to serum calcium trajectory analysis, group comparisons, and multivariable model construction and validation. BMI, body-mass index; GCS, Glasgow Coma Scale; LASSO, Least Absolute Shrinkage and Selection Operator; ROC, receiver operating characteristic; SAH, spontaneous subarachnoid hemorrhage.

This single-center observational cohort study with standardized protocols was conducted at Tianjin Medical University General Hospital (TMUGH). All consecutive adults with SAH admitted between 1 January 2018 and 1 February 2025 were screened according to the predefined clinical eligibility criteria; all patients who satisfied these criteria were enrolled and followed for 90 ± 14 days. Inclusion criteria were: (1) age 18–80 years; (2) SAH verified by non-contrast cranial CT and further characterized by CT angiography or digital subtraction angiography within 24 h of ictus; (3) admission and initiation of conservative treatment within 24 h after symptom onset; (4) availability of serial serum calcium measurements at admission, 72 h ± 6 h and 7 days ± 1 day, together with complete demographic and clinical data; and (5) completion of neuroimaging follow-up at 90 ± 14 days. Exclusion criteria were: (1) onset-to-admission interval > 3 days; (2) co-existing intracranial disease such as tumors or additional hemorrhage; (3) traumatic SAH or hemorrhage secondary to arteriovenous malformation, moyamoya disease or other non-aneurysmal causes; (4) pre-existing neurological deficit, coagulation disorders, severe hepatic/renal dysfunction, or systemic illnesses known to disturb calcium homoeostasis; (5) Patients who received calcium supplementation during hospitalization, which could confound the measurement of serum calcium levels; and (6) missing key clinical or laboratory information. Accordingly, patients who could not complete the prespecified serial serum calcium measurements (admission, 72 h, and 7 days) were not eligible for inclusion, and patients without 90 ± 14-day follow-up neuroimaging were considered not evaluable for the primary outcome (Figure 1). The protocol was approved by the TMUGH Ethics Committee (Approval No. IRB2025-YX-262-01); the committee granted a waiver of written consent because all blood sampling and follow-up imaging formed part of routine clinical care. The datasets generated and/or analyzed during the current study are not publicly available due to patient privacy and institutional data protection policies. However, they may be obtained from the corresponding author upon reasonable request, subject to approval by the Tianjin Medical University General Hospital Ethics Committee and completion of a data use agreement.

### Data collection

Baseline information—mean age, sex, body-mass index (BMI), and admission systolic/diastolic blood pressure—was extracted from the electronic medical record together with documented comorbidities (prior stroke, hepatic dysfunction), cardiovascular risk factors (hypertension, diabetes, dyslipidemia, alcohol abuse, smoking), and chronic drug use (anticoagulants, antiplatelet agents, antihypertensives). We also recorded in-hospital course variables—admission serum sodium, hyponatremia during hospitalization (Na < 135 mmol/L), and nimodipine exposure. In addition, serum calcium measurements at each time point (admission, 72 h, and 7 days) were categorized by the presence or absence of in-hospital hyponatremia (Na < 135 mmol/L). Neurological severity on admission was independently assessed by two neurosurgeons using the GCS and Hunt-Hess grade. At each predefined time point (admission [T0], 72 h ± 6 h [T1], and 7 days ± 1 day [T2]), 2 mL of peripheral venous blood was collected, allowed to clot, and centrifuged at 4000 rpm for 5 min to obtain serum. Serum samples were aliquoted immediately after centrifugation and stored at 4 °C, and calcium measurements were completed within 2 h. Serum calcium concentrations were measured in duplicate using an automated o-cresolphthalein-complexone colorimetric assay (AU5800 Chemistry Analyzer, Beckman Coulter, Brea, CA, USA), with an inter-assay coefficient of variation of less than 3%.

### Definition of delayed hydrocephalus

**Figure 2:**
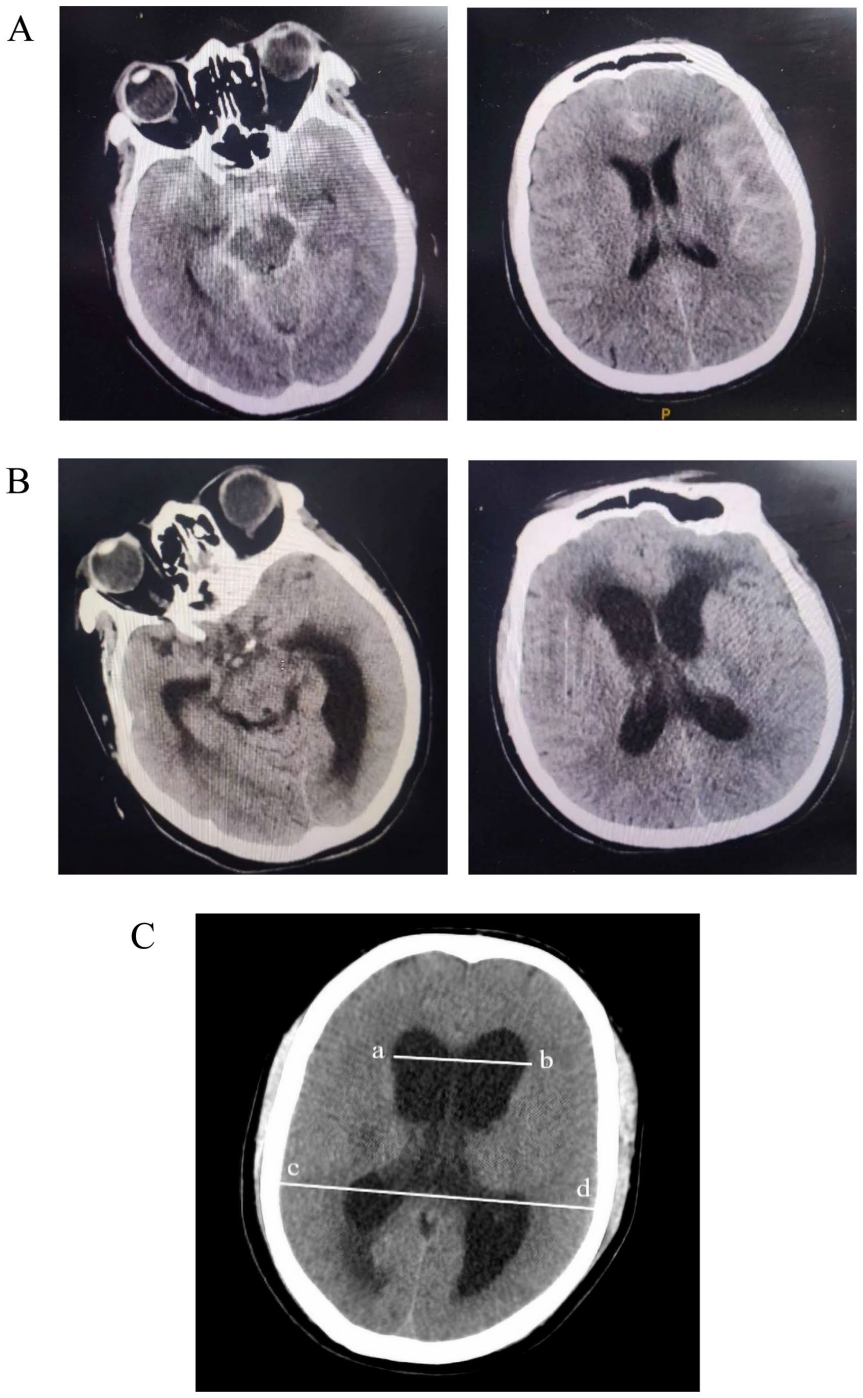
Radiological definition of delayed hydrocephalus. **(A)** CT image on admission showing typical subarachnoid hemorrhage without ventricular enlargement. **(B)** Follow-up CT at 90 days showing progressive ventricular dilation. Red arrows indicate progressive ventricular dilation on follow-up CT images. **(C)** Illustration of the Evans Index, calculated as the ratio of the maximum width of the frontal horns (line a, b) to the maximum internal diameter of the skull (line c, d). An Evans Index > 0.30 indicates radiological hydrocephalus.

The primary outcome of delayed hydrocephalus was diagnosed within 90 ± 14 days when both of the following criteria were met: (1) radiological enlargement of the ventricular system, defined as an Evans Index > 0.30 or a biventricular width ≥ 45 mm on CT/MRI, and (2) new or progressive neurological deterioration that prompted cerebrospinal-fluid diversion (external ventricular or lumbar drainage, or ventriculoperitoneal shunting). Figures 2A,B show typical CT findings of SAH and ventricular enlargement at admission and 3-month follow-up, respectively. Arrows in Figure 2B highlight the regions of progressive ventricular enlargement on the follow-up CT images. Figure 2C illustrates the Evans Index, calculated as the ratio of the maximum distance between the anterior horn of the lateral ventricle (line a-b) to the maximum cranial diameter (line c-d), with the formula: Evans Index = ab/cd. Two board-certified neuroradiologists, blinded to laboratory data, independently adjudicated each case; disagreements were resolved by consensus.

### Longitudinal serum calcium trajectories analysis

To elucidate the dynamic profile of serum calcium following SAH and its relationship with subsequent delayed hydrocephalus, we conducted a multi-step longitudinal analysis. First, serum calcium concentrations measured at admission (T0, within 24 h post-ictus), 72 h ± 6 h (T1), and 7 days ± 1 day (T2) were compared across all patients using repeated measures ANOVA or, for non-normally distributed data, the Friedman test; *post hoc* pairwise comparisons employed Bonferroni correction. This approach characterized overall temporal trends and identified significant shifts in calcium levels over the acute phase.

The relationship between calcium fluctuations and neurological severity was assessed through longitudinal correlation analysis. Changes in serum calcium concentrations were correlated with variations in GCS scores and Hunt-Hess grades using Pearson’s or Spearman’s correlation coefficients, depending on normality assumptions. This analysis aimed to determine whether early biochemical alterations were linked to clinical deterioration.

To investigate the potential of serum calcium as a predictive biomarker for delayed hydrocephalus, patients were stratified into Delayed Hydrocephalus and Non-Delayed Hydrocephalus groups based on predefined radiological and clinical criteria at 90 ± 14 days. Dynamic changes in serum calcium for each group were plotted and compared at T0, T1, and T2 using independent-samples *t* tests or Mann–Whitney U tests, as appropriate, to detect inter-group differences at each time point.

Predictive performance was further evaluated using receiver operating characteristic (ROC) curve analysis. Serum calcium levels at each time point, along with admission GCS scores and Hunt-Hess grades, were tested for their ability to discriminate patients who developed delayed hydrocephalus. The area under the ROC curve (AUC), sensitivity, specificity, positive predictive value (PPV), and negative predictive value (NPV) were calculated for each variable. Optimal cutoff values were determined using Youden’s index (sensitivity + specificity − 1) from the ROC curves.

To further evaluate the risk stratification capability of serum calcium, patients were categorized into quartile groups (Q1–Q4) at each time point based on interquartile ranges (IQR) of serum calcium. The incidence rates of delayed hydrocephalus within each quartile were calculated, and trend analyses were conducted to determine whether specific quartiles were associated with increased or decreased delayed hydrocephalus risk. These analyses aimed to establish whether dynamic serum calcium monitoring could serve as a practical tool for early identification of high-risk patients following SAH. To translate these findings into a clinically actionable framework, we further proposed a preliminary clinical decision pathway for delayed hydrocephalus risk stratification and early management in SAH patients, integrating calcium-based thresholds with neurological severity scores to support individualized surveillance and intervention strategies during the acute phase.

### Admission-variable prediction model

To develop a predictive model for delayed hydrocephalus following SAH, we first identified clinical variables available at admission, including serum calcium concentration and other baseline indicators. Univariate analysis was performed in the overall cohort to identify variables significantly associated with delayed hydrocephalus (*p* < 0.05). Variables meeting this criterion were selected as candidate predictors for further modeling.

The dataset was then randomly split into a training cohort (70%) and an internal hold-out validation cohort (30%). In the training cohort, Least Absolute Shrinkage and Selection Operator (LASSO) regression was applied to the candidate variables to reduce dimensionality and address multicollinearity. The optimal penalization parameter (*λ*) was determined using 10-fold cross-validation, selecting the λ value corresponding to one standard error (λ-1SE) of the minimum deviance. Variables with non-zero coefficients were retained and entered into a multivariable logistic regression model. Based on the final model, a nomogram was constructed to provide individualized risk estimation for delayed hydrocephalus.

Model performance was evaluated at three levels. (i) In the 70% training cohort, beyond resampling-based internal validation with bootstrap resampling (1,000 iterations) and k-fold cross-validation to assess stability and potential optimism, we also reported apparent performance in the same prespecified sequence: first, the confusion matrix with threshold-specific classification indices (sensitivity, specificity, PPV, NPV); next, discrimination by ROC curves with AUC and 95% confidence intervals (CI) together with precision-recall (PR) curves; then calibration by calibration plots with the calibration intercept and slope and the Hosmer-Lemeshow (HL) goodness-of-fit test; and finally, clinical utility by decision-curve analysis (DCA) across clinically relevant thresholds. (ii) In the 30% internal validation cohort, model evaluation followed the identical order—confusion matrix and threshold-level indices, ROC/AUC (95% CIs) and PR curves, calibration plots with calibration intercept and slope (plus HL testing), and DCA. (iii) External validation: we retrospectively assembled an independent external cohort from the Departments of Neurosurgery at the Affiliated Hospital of Qinghai University and the North China University of Science and Technology Affiliated Hospital, restricted to patients with complete admission data for the final set of predictors used in the nomogram (i.e., those retained after LASSO). Consecutive eligible admissions were identified between January 1, 2023, and February 1, 2025. The primary outcome (delayed hydrocephalus within 90 ± 14 days) was ascertained via structured telephone follow-up with the patient or a primary caregiver, supplemented by source verification in the hospital information system (readmissions and discharge/operative notes indicating CSF diversion), outpatient/neurosurgical clinic records, and central blinded review of follow-up CT/MRI according to a harmonized protocol; if unreachable by phone, at least two additional attempts at different times and an alternative proxy were contacted. These external cases were used solely for external validation without model refitting; performance for the pooled external cohort was reported using the same sequence—confusion matrix with threshold-level indices, ROC/AUC (95% CIs) and PR curves, calibration plots with calibration intercept and slope (plus HL testing), and DCA.

### Statistical analysis

All statistical analyses were conducted using SPSS Statistics version 25.0 (IBM Corp., Armonk, NY, USA), Python version 3.10,^1^ and R version 4.2.0.^2^ Continuous variables were tested for normality and expressed as means with standard deviations or medians with IQRs, and categorical variables were reported as frequencies and percentages. Between-group comparisons used the t test or Mann–Whitney U test for continuous variables and the chi-square test or Fisher’s exact test for categorical variables, as appropriate; longitudinal comparisons across time points used repeated-measures ANOVA or the Friedman test with Bonferroni-adjusted *post hoc* analyses. Missing data were handled using MICE. The imputation model included all variables listed in Table 1, and 5 imputed datasets were generated; the outcome was not imputed. Robustness was assessed by comparing observed vs. imputed distributions. Hypothesis testing was two-tailed, and a *p*-value < 0.05 was considered statistically significant.

**Table 1:** Baseline characteristics of 302 patients with spontaneous subarachnoid hemorrhage: comparison between Non-Delayed Hydrocephalus and Delayed Hydrocephalus groups.

Variable	Total (*n* = 302)	Non-Delayed Hydrocephalus Group (*n* = 229)	Delayed Hydrocephalus Group (*n* = 73)	*χ*^2^/*t*-value	*p* value
Age (years)	69.6 ± 13.4	69.1 ± 12.7	70.3 ± 14.2	0.580	0.563
Sex (*n*, %)					
Male	186 (61.6)	142 (62.0)	44 (60.3)	0.123	0.731
Female	116 (38.4)	87 (38.0)	29 (39.7)		
BMI (kg/m^2^)	24.1 ± 3.8	23.9 ± 3.7	24.6 ± 4.0	1.348	0.179
Blood pressure (mmHg)					
Systolic pressure	178.1 ± 30.3	175.5 ± 29.4	187.6 ± 31.7	3.558	<0.001
Diastolic pressure	95.8 ± 18.7	94.3 ± 18.0	103.1 ± 19.2	4.178	<0.001
Comorbidities (*n*, %)					
History of stroke	68 (22.5)	51 (22.3)	17 (23.3)	0.122	0.731
Hepatic dysfunction	31 (10.3)	24 (10.5)	7 (9.6)	0.268	0.620
Risk factors (*n*, %)					
Hypertension	211 (69.9)	146 (63.8)	65(89.6)	6.358	<0.001
Diabetes	74 (24.5)	44 (19.2)	30 (41.1)	10.765	0.031
Dyslipidemia	58 (19.3)	21 (9.3)	37 (50.6)	9.547	0.005
Alcohol use	144 (47.7)	82 (35.8)	62 (85.4)	2.549	<0.001
Smoking history	69 (22.9)	41 (17.9)	28 (38.7)	0.814	0.024
Medications (*n*, %)					
Anticoagulants	16 (5.3)	13 (5.7)	3 (4.1)	0.318	0.573
Antiplatelet agents	22 (7.3)	17 (7.4)	5 (6.8)	0.141	0.708
Antihypertensives	32 (10.6)	25 (10.9)	7 (9.6)	0.122	0.731
In-hospital course					
Serum sodium at admission (mmol/L)	140.4 ± 4.6	140.8 ± 4.8	138.5 ± 5.5	1.750	0.080
Hyponatremia during hospitalization (*n*, %)	106(35.1)	79(34.5)	27(37.0)	0.150	0.700
Nimodipine use during hospitalization (*n*, %)	299(99.0)	227(99.1)	72(98.6)	0.140	0.710
Neurological function (score)					
GCS score	9.6 ± 3.8	10.2 ± 3.1	7.1 ± 3.9	8.316	<0.001
Hunt-Hess grade	2.7 ± 1.5	2.4 ± 1.4	3.4 ± 1.6	6.449	<0.001

*χ*^2^/*t*-value and *p*-value represent the comparison between Non-Delayed Hydrocephalus and Delayed Hydrocephalus groups.

BMI, body-mass index; GCS, Glasgow Coma Scale.

## Results

### Baseline characteristics of the cohort

A total of 340 patients were enrolled in the study. Among these, 302 (88.8%) completed the 90 ± 14 days neuroimaging follow-up and were included in the primary outcome analysis. The remaining 38 patients (11.2%) were not evaluable for the primary outcome, including 18 who died before the follow-up assessment, 12 in whom follow-up imaging was unavailable, and 8 who were unable to be contacted or refused follow-up (Figure 1). Baseline data were available for all 340 patients, with >98% completeness across all variables. Missing values were addressed using MICE.

The enrolled population had a mean ± SD age of 69.6 ± 13.4 years and was predominantly male (186/302, 61.6%). The mean BMI was 24.1 ± 3.8 kg/m^2^. Arterial hypertension was prevalent among participants: the mean admission systolic/diastolic blood pressures were 178.1 ± 30.3/95.8 ± 18.7 mmHg, and 211 patients (69.6%) had a prior history of hypertension. Other cardiovascular or metabolic risk factors included diabetes (24.5%), dyslipidemia (19.3%), habitual alcohol use (47.7%), and current smoking (22.9%). A history of stroke and hepatic dysfunction was present in 22.5% and 10.3% of patients, respectively. Chronic use of anticoagulants, antiplatelet agents, and antihypertensives was documented in 5.3%, 7.3%, and 10.6% of patients, respectively. During hospitalization, hyponatremia occurred in 35.1% of patients, and nearly all patients (99.0%) received nimodipine. Neurological status on admission was generally poor, with a mean GCS of 9.6 ± 3.8 and a mean Hunt-Hess grade of 2.7 ± 1.5. The overall study design is illustrated in Figure 1, and detailed baseline characteristics are summarized in Table 1.

### Temporal profile of serum calcium and its correlation with initial severity

**Figure 3:**
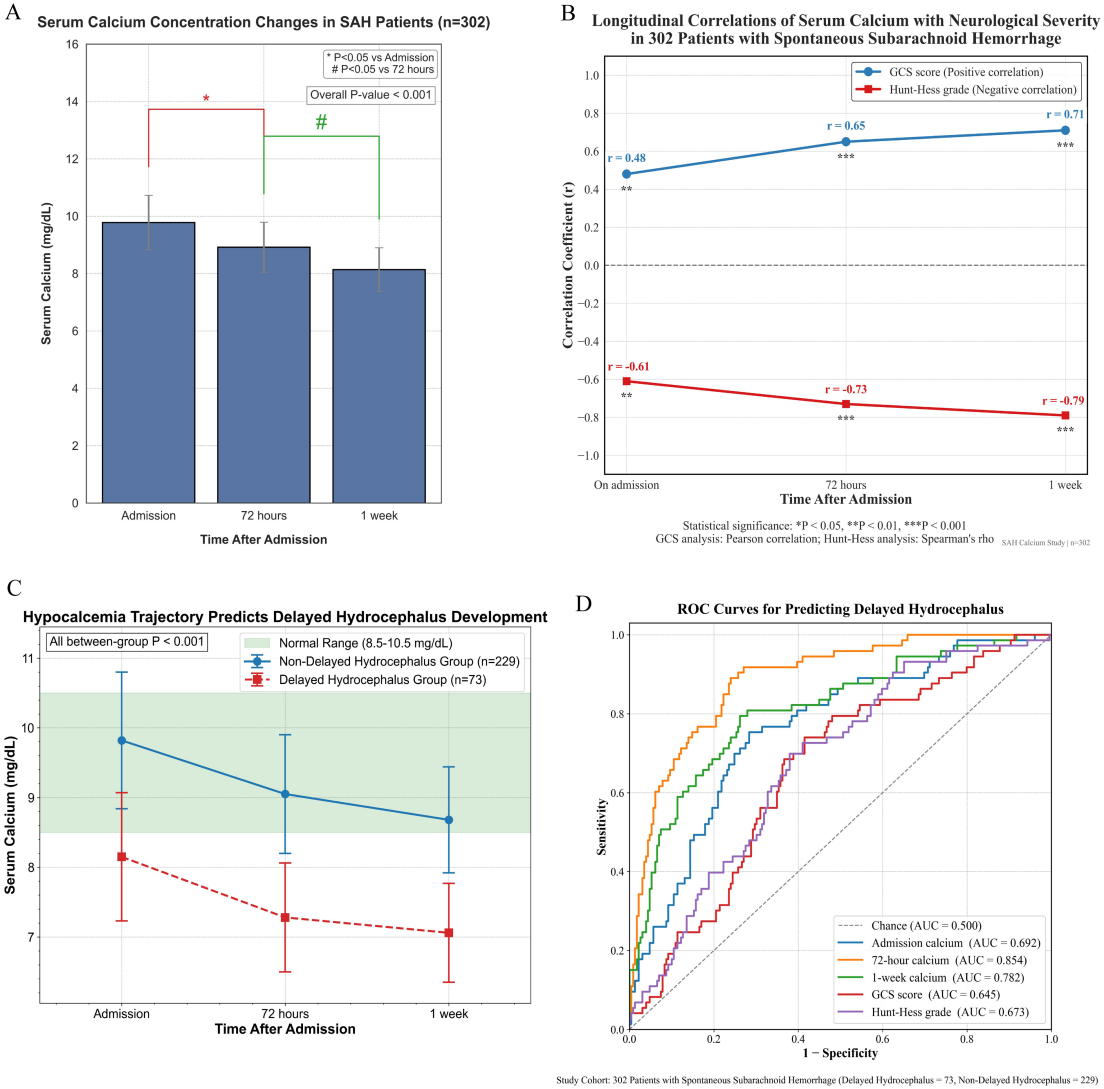
Temporal trends and predictive value of serum calcium in SAH patients. **(A)** Serum calcium levels progressively declined from admission to day 7 following ictus (*p* < 0.001). **(B)** Serum calcium showed positive correlation with GCS and negative correlation with Hunt-Hess grade across time points. **(C)** Compared to non-delayed hydrocephalus patients, those who developed delayed hydrocephalus had significantly lower serum calcium at all time points (*p* < 0.001). **(D)** ROC analysis demonstrated that calcium levels at 72 h had the highest predictive value for delayed hydrocephalus (AUC = 0.854), outperforming neurological scores and other time points. GCS, Glasgow Coma Scale; ROC, receiver operating characteristic; SAH, spontaneous subarachnoid hemorrhage.

Serum calcium levels exhibited a progressive decline over the first week following ictus (Figure 3A; Supplementary Table S1). The mean concentration at admission was 9.78 ± 0.95 mg/dL, which decreased to 8.92 ± 0.87 mg/dL at 72 h (*p* < 0.05 vs. admission) and further declined to 8.14 ± 0.76 mg/dL at day 7 (*p* < 0.05 vs. both earlier time points). Repeated-measures ANOVA confirmed a significant temporal effect across these time points (*p* < 0.001).

Furthermore, lower serum calcium levels were consistently associated with worse neurological status throughout the acute phase. At admission, serum calcium correlated positively with GCS score (*r* = 0.48, *p* = 0.009) and negatively with Hunt-Hess grade (*r* = −0.61, *p* = 0.003). These associations strengthened over time, with correlation coefficients reaching *r* = 0.71 (GCS) and *r* = −0.79 (Hunt-Hess) by day 7 (all *p* < 0.001) (Figure 3B; Supplementary Table S2). These findings suggest that early hypocalcemia not only reflects but also tracks with the severity of neurological injury during the acute phase of SAH.

### Incidence of delayed hydrocephalus and baseline comparison between groups

During the 90-day follow-up, 73 out of 302 patients (24.2%) developed delayed hydrocephalus. Baseline characteristics of patients with and without delayed hydrocephalus are summarized in Table 1. No significant differences were observed between groups in terms of age, sex, BMI, history of stroke, hepatic dysfunction, or chronic use of anticoagulants, antiplatelet agents, or antihypertensives (all *p* > 0.10).

In contrast, the Delayed Hydrocephalus group exhibited significantly higher systolic (187.6 ± 31.7 vs. 175.5 ± 29.4 mmHg) and diastolic (103.1 ± 19.2 vs. 94.3 ± 18.0 mmHg) blood pressures on admission (both *p* < 0.001). Vascular and metabolic comorbidities were also more prevalent in the Delayed Hydrocephalus group, including hypertension (89.6% vs. 63.8%), diabetes (41.1% vs. 19.2%), dyslipidemia (50.6% vs. 9.3%), alcohol abuse (85.4% vs. 35.8%), and current smoking (38.7% vs. 17.9%), with all comparisons reaching statistical significance (*p* ≤ 0.031).

Neurological status at presentation was markedly poorer among patients who later developed delayed hydrocephalus. Their mean GCS score was significantly lower (7.1 ± 3.9 vs. 10.2 ± 3.1, *p* < 0.001), while their Hunt-Hess grades were significantly higher (3.4 ± 1.6 vs. 2.4 ± 1.4, *p* < 0.001), indicating more severe initial brain injury.

### Dynamic changes in serum calcium and diagnostic performance for delayed hydrocephalus

Dynamic changes in serum calcium showed a decline over time in both Delayed Hydrocephalus and Non-Delayed Hydrocephalus groups but remained consistently lower in patients who eventually developed delayed hydrocephalus (Figure 3C; Supplementary Table S3). At admission, the mean serum calcium level was significantly reduced in the Delayed Hydrocephalus group compared to the Non-Delayed Hydrocephalus group (8.15 ± 0.92 vs. 9.82 ± 0.98 mg/dL; *p* < 0.001). This difference persisted at 72 h (7.28 ± 0.78 vs. 10.35 ± 2.11 mg/dL; *p* < 0.001) and at 1 week (7.06 ± 0.71 vs. 8.88 ± 1.57 mg/dL; *p* < 0.001). Repeated-measures ANOVA confirmed a significant group × time interaction (*p* < 0.001), indicating a steeper early decline in serum calcium among patients who developed delayed hydrocephalus.

ROC curve analysis demonstrated that serum calcium measured at 72 h provided the highest discriminatory power for predicting delayed hydrocephalus (AUC = 0.854, 95% CI: 0.822–0.886). At the optimal cutoff of ≤7.65 mg/dL, this parameter yielded a sensitivity of 85.6%, specificity of 80.3%, PPV of 72.8%, and NPV of 90.6% (Table 2; Figure 3D). In comparison, predictive accuracy was lower for admission calcium (AUC = 0.692) and 1-week calcium (AUC = 0.782). Admission GCS (AUC = 0.645) and Hunt-Hess grade (AUC = 0.673) were also significant but exhibited weaker predictive performance.

**Table 2:** ROC analysis of predictors for delayed hydrocephalus after spontaneous subarachnoid hemorrhage.

Predictor	AUC (95% CI)	Cut-off value	*p*-value	Sensitivity (%)	Specificity (%)	PPV (%)	NPV (%)	Youden index
Admission calcium	0.692 (0.648–0.736)	≤8.75 mg/dL	<0.001	72.6	73.8	59.3	83.7	0.464
72-h calcium	0.854 (0.822–0.886)	≤7.65 mg/dL	<0.001	85.6	80.3	72.8	90.6	0.659
1-week calcium	0.782 (0.742–0.822)	≤7.45 mg/dL	<0.001	79.5	75.1	65.2	86.3	0.546
GCS score	0.645 (0.600–0.690)	≤8.00	0.018	73.9	60.7	49.8	81.2	0.346
Hunt-Hess grade	0.673 (0.628–0.718)	≥3.00	0.009	70.4	65.1	51.6	80.3	0.355

AUC, area under the curve; CI, confidence interval; GCS, Glasgow Coma Scale; NPV, negative predictive value; PPV, positive predictive value.

**Figure 4:**
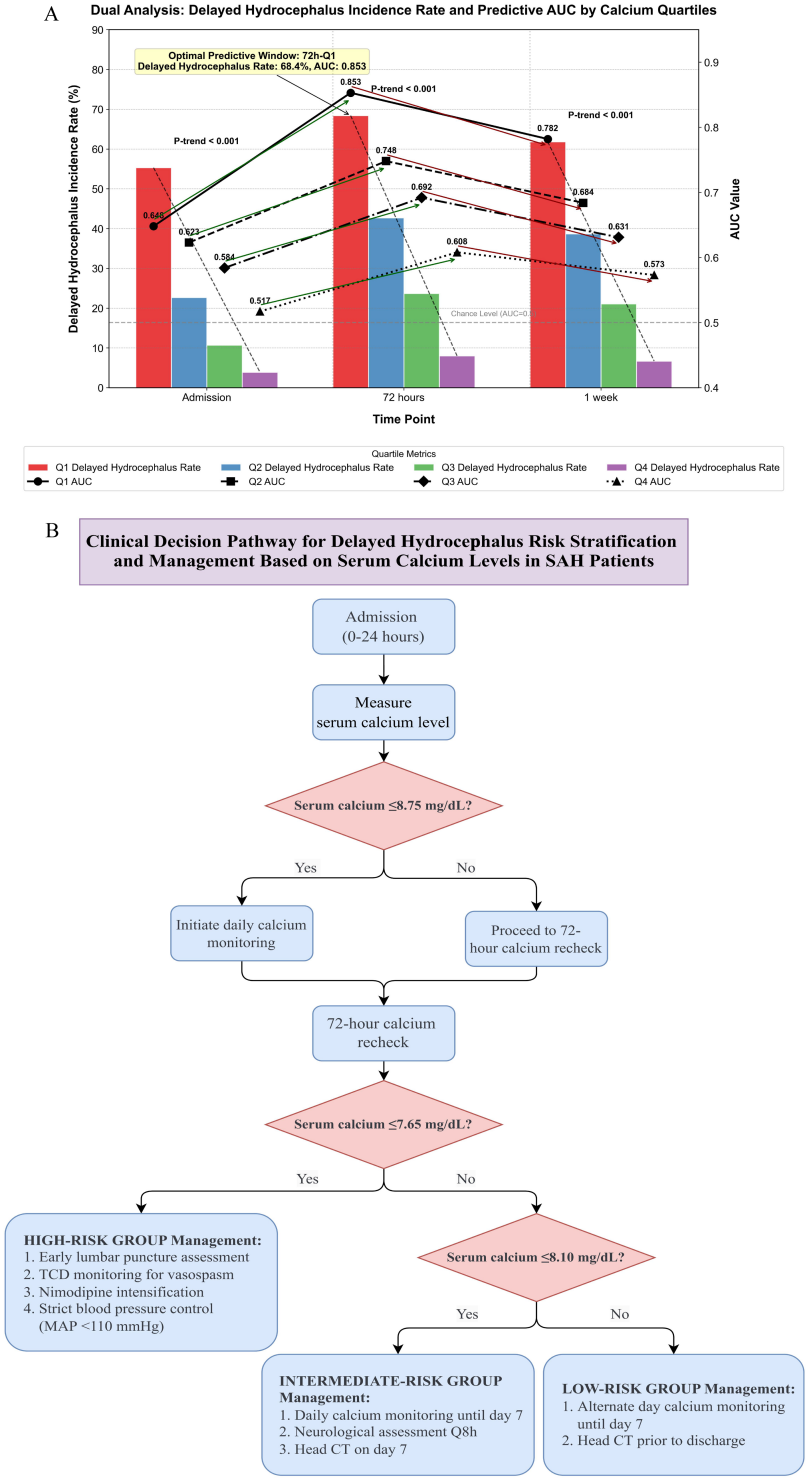
Quartile-based serum calcium analysis and calcium-guided risk pathway for delayed hydrocephalus after spontaneous SAH. **(A)** Delayed hydrocephalus incidence (bars, %) and predictive performance (AUC; lines) across serum calcium quartiles at admission, 72 h, and 1 week. Lower calcium consistently associated with higher delayed hydrocephalus risk; the 72-h lowest quartile (≤7.65 mg/dL) showed peak incidence (≈68%) and highest AUC (0.853). **(B)** Proposed clinical decision pathway using calcium thresholds (admission ≤8.75 mg/dL; 72-h ≤ 7.65 mg/dL; day-7 ≤ 8.10 mg/dL) to classify high-, intermediate-, and low-risk groups and guide monitoring and management. AUC, area under the curve; CT, computed tomography; MAP, mean arterial pressure; TCD, transcranial Doppler ultrasonography SAH, spontaneous subarachnoid hemorrhage.

To further explore the dose–response relationship between calcium and delayed hydrocephalus risk, patients were stratified into quartiles (Q1–Q4) based on serum calcium levels at admission, 72 h, and 1 week (Supplementary Table S4). As illustrated in Figure 4A, delayed hydrocephalus incidence decreased progressively across quartiles at all time points (P-trend < 0.001). The strongest association was observed at 72 h, where the lowest quartile (Q1, ≤7.65 mg/dL) showed a delayed hydrocephalus incidence of 68.4%, while the highest quartile (Q4, ≥8.66 mg/dL) had an incidence of only 8.0%. Correspondingly, AUC values followed a descending trend across quartiles—from 0.853 in Q1 to 0.608 in Q4—highlighting both a clear stratification pattern and the limited utility of higher calcium ranges for prediction. Similar trends were evident at admission and 1 week, though with lower overall predictive power.

Building upon these findings, we developed a preliminary risk stratification pathway to guide individualized management of delayed hydrocephalus based on serum calcium trajectories (Figure 4B). This flowchart incorporates two key thresholds (≤8.75 mg/dL at admission and ≤7.65 mg/dL at 72 h), enabling early classification into high-, intermediate-, or low-risk groups. Corresponding clinical recommendations include lumbar puncture evaluation, intensified vasospasm surveillance, tailored imaging schedules, and frequency-adjusted calcium monitoring protocols. Such stratification may facilitate timely interventions and improve outcomes in SAH patients at high risk of hydrocephalus.

### Association between in-hospital hyponatremia and serum calcium levels

Serial serum calcium levels were compared between patients with and without in-hospital hyponatremia to assess for potential confounding. No significant differences were found at any time point (admission, 72 h, or 7 days; Supplementary Table S5), and the magnitude of the early calcium decline was similar between groups. This indicates that the hypocalcemia predictive of hydrocephalus is independent of hyponatremia status.

### Admission-variable prediction model

Although serum calcium measured at 72 h exhibited the strongest association with delayed hydrocephalus, its availability is inherently delayed, limiting its clinical utility for early prognostication. To enable timely risk assessment at admission, we developed a predictive model based exclusively on baseline variables.

**Figure 5:**
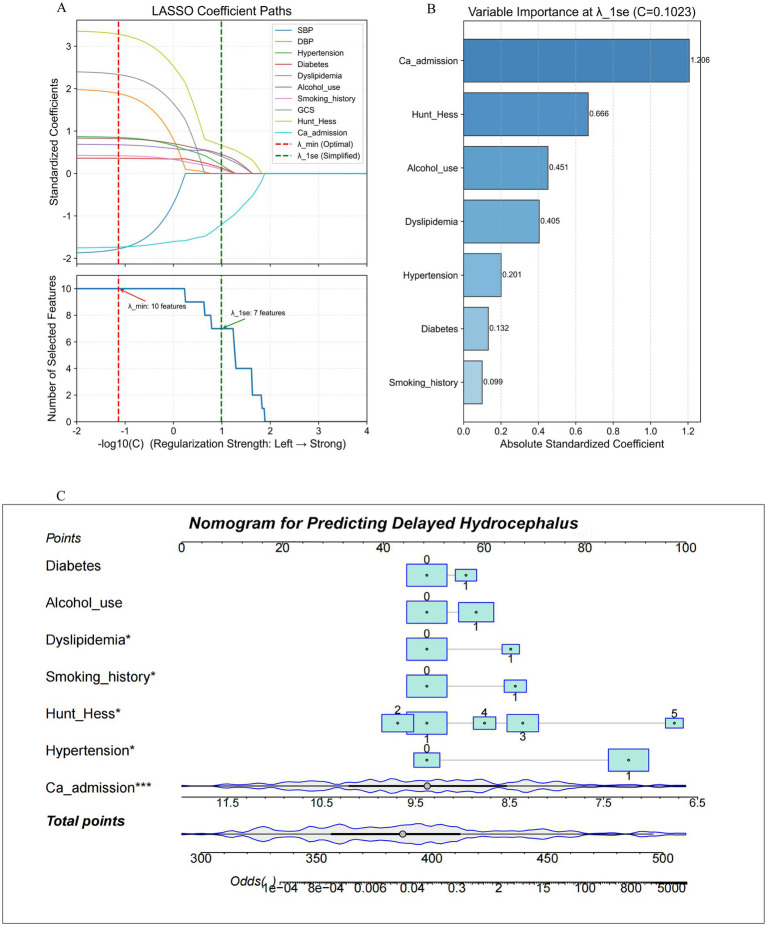
Admission-based prediction model for delayed hydrocephalus after SAH. **(A)** LASSO regression with 10-fold cross-validation identifying predictors at the *λ*-1SE threshold. **(B)** Standardized coefficients of the seven variables retained: admission serum calcium, Hunt-Hess grade, alcohol use, dyslipidemia, hypertension, diabetes, and smoking history. **(C)** Nomogram derived from multivariable logistic regression to estimate individual delayed hydrocephalus risk at admission. Asterisks indicate statistical significance of predictors in the multivariable logistic regression model (two-sided Wald test): * *p* < 0.05; *** *p* < 0.001. DBP, diastolic blood pressures; LASSO, least absolute shrinkage and selection operator; SAH, spontaneous subarachnoid hemorrhage; SBP, systolic diastolic blood pressures.

Using the LASSO method with 10-fold cross-validation, seven variables were selected at the *λ*-1SE threshold: admission serum calcium, Hunt-Hess grade, alcohol use, dyslipidemia, hypertension, diabetes, and smoking history (Figure 5A). Their relative importance is illustrated by the standardized coefficients in Figure 5B.

These seven variables were incorporated into a logistic regression model using the training set (*n* = 211), and a nomogram was subsequently developed as a user-friendly clinical tool (Figure 5C). In practice, clinicians can assign a specific score to each variable based on the patient’s baseline profile—for example, the presence of diabetes yields 56 points. The total score, obtained by summing individual contributions, maps directly to a predicted probability of developing delayed hydrocephalus. A higher total score indicates a correspondingly greater risk.

**Figure 6:**
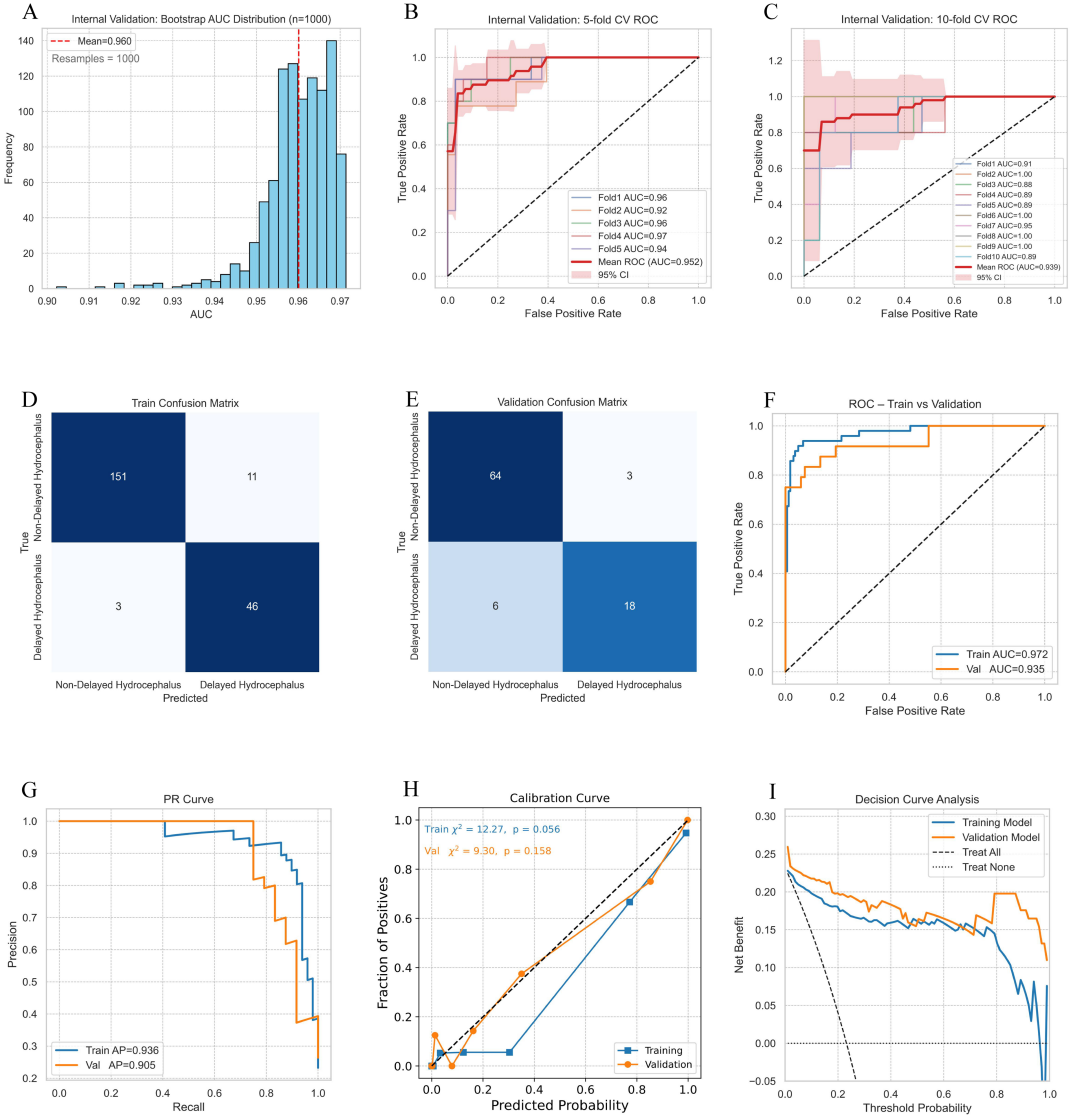
Internal validation of the predictive model for delayed hydrocephalus. **(A)** Bootstrap resampling (*n* = 1,000) demonstrating a narrow AUC distribution with a mean of 0.960, indicating model stability. **(B,C)** 5-fold and 10-fold cross-validation showing consistently high AUCs (5-fold AUC = 0.952, 10-fold AUC = 0.939), reinforcing model robustness with minimal overfitting. **(D,E)** Confusion matrix with threshold-level classification performance: training sensitivity = 93.9%, specificity = 93.2%, PPV = 80.7%, NPV = 98.1%; validation sensitivity = 75.0%, specificity = 95.5%, PPV = 85.7%, NPV = 91.4%. **(F)** ROC curve analysis showing strong discrimination with training ROC AUC = 0.972 and validation ROC AUC = 0.935. **(G)** PR curve analysis with training PR-AUC = 0.936 and validation PR-AUC = 0.905. **(H)** Calibration plot showing good agreement between predicted and observed risks, with training HL *p* = 0.056 and validation HL *p* = 0.158. **(I)** Decision curve analysis indicating clear net clinical benefit across various threshold probabilities. AUC, area under the curve; DCH, delayed hydrocephalus; NPV, negative predictive value; PPV, positive predictive value; PR, precision-recall; ROC, receiver operating characteristic.

Internal validation proceeded in two steps. First, in the 70% training cohort, bootstrap resampling (*n* = 1,000) yielded a narrow AUC distribution with a mean of 0.960 (Figure 6A). Both 5-fold and 10-fold cross-validation showed consistently high AUCs (5-fold AUC = 0.952, 10-fold AUC = 0.939) across folds (Figures 6B,C), indicating stable discrimination with minimal overfitting. In addition to these methods, we also evaluated apparent performance: the confusion matrix showed favorable threshold-level classification performance with training sensitivity = 93.9%, specificity = 93.2%, PPV = 80.7%, and NPV = 98.1% (Figures 6D); and validation sensitivity = 75.0%, specificity = 95.5%, PPV = 85.7%, and NPV = 91.4% (Figure 6E). Discrimination was strong, as evidenced by training ROC AUC = 0.972 and validation ROC AUC = 0.935 (Figures 6F); and training PR-AUC = 0.936 and validation PR-AUC = 0.905 (Figure 6G). Calibration demonstrated good agreement between predicted and observed risk, with training HL *p* = 0.056 (Figure 6H) and validation HL *p* = 0.158. Finally, DCA indicated net clinical benefit across clinically relevant threshold probabilities (Figure 6I).

For external validation, The locked model (no refitting) was evaluated in a pooled external test cohort consisting of 142 patients aggregated from two hospitals: 88 patients from the North China University of Science and Technology Affiliated Hospital (completed follow-up: 78, DCH: 19, non-DCH: 59) and 72 patients from the Affiliated Hospital of Qinghai University (completed follow-up: 64, DCH: 15, non-DCH: 49) (Supplementary Table S6). Supplementary Figure S1 illustrates the external validation results for the model in this cohort. The external validation model showed excellent discriminatory power with a ROC AUC of 0.898 and a PR AUC of 0.739, demonstrating good generalizability when applied to the independent cohort from two hospitals. Sensitivity, specificity, PPV, and NPV were robust across clinically relevant thresholds. Calibration was acceptable, with a HL test *p*-value of 0.061, and DCA confirmed significant net clinical benefit at multiple threshold levels.

## Discussion

This study demonstrates that dynamic changes in serum calcium carry significant prognostic value for delayed hydrocephalus after SAH. In this clinically monitored observational cohort of 302 SAH patients, lower serum calcium levels—particularly at 72 h post-ictus—were more strongly associated with subsequent delayed hydrocephalus development than conventional neurological scores such as the Hunt-Hess grade or admission GCS. A clear dose–response relationship was observed across calcium quartiles, supporting the feasibility of threshold-based biochemical risk stratification. Beyond dynamic serum calcium monitoring, we constructed a nomogram based solely on admission variables—including serum calcium, Hunt-Hess grade, and vascular risk factors—which demonstrated strong predictive performance. This tool enables individualized risk estimation at the bedside using routine clinical information, offering early decision support before 72-h laboratory data become available. Together, these findings establish both static (admission-based) and dynamic (serial calcium-based) serum calcium assessments as complementary strategies for early delayed hydrocephalus prediction and intervention planning in clinical practice.

The observed association between early hypocalcemia and delayed hydrocephalus development is consistent with emerging evidence implicating calcium dysregulation in secondary brain injury after SAH. Calcium serves as a vital intracellular messenger that regulates neuronal excitability, synaptic transmission, BBB integrity, and cell survival (23–26). Following aneurysmal rupture, a surge of vasoactive substances—including endothelin-1, serotonin, and thromboxane A2—activates calcium channels, resulting in pathological intracellular calcium overload (27, 28). This cascade exacerbates cerebral vasospasm, mitochondrial dysfunction, neuroinflammation, and glial scar formation, ultimately disrupting CSF circulation and predisposing to hydrocephalus (29, 30). In our study, the decline in serum calcium was more marked and persistent among patients who developed delayed hydrocephalus, suggesting a prolonged disturbance in systemic calcium regulation, possibly driven by acute stress responses, inflammatory cytokines, or suppression of parathyroid hormone. Importantly, we do not interpret serum calcium as a direct measure of intracellular Ca^2+^ overload; rather, it is an easily obtainable biomarker that may capture systemic disturbances in calcium regulation and illness severity that accompany SAH. Furthermore, prior studies have shown that hypocalcemia weakens endothelial tight junctions, increasing BBB permeability and promoting the infiltration of inflammatory mediators into the brain parenchyma (31). These changes may contribute to periventricular gliosis and impaired CSF absorption—hallmarks of post-SAH hydrocephalus. Taken together, these mechanistic insights support the hypothesis that hypocalcemia is not merely a passive bystander or severity marker, but an active participant in the pathophysiological cascade leading to delayed hydrocephalus. Our findings offer new clinical evidence reinforcing this concept and highlight the potential of serum calcium as both a biomarker and a modifiable target in post-SAH care.

The strong predictive performance of 72-h serum calcium highlights its clinical utility as a practical risk stratification marker for delayed hydrocephalus in SAH patients. Unlike many biomarkers that require specialized assays or delayed radiological findings, serum calcium is widely available, inexpensive, and rapidly obtainable across all care settings. In our cohort, a 72-h threshold of ≤7.65 mg/dL effectively identified high-risk patients, while those with higher calcium levels exhibited a substantially lower likelihood of delayed hydrocephalus development. These early biochemical signals can inform real-time decision-making—prompting intensified surveillance, modified imaging intervals, or preemptive interventions such as early lumbar puncture or targeted vasospasm management in high-risk individuals (32, 33). Moreover, the proposed pathway based on dynamic serum calcium monitoring offers a resource-efficient framework for personalized care. By categorizing patients into high-, intermediate-, and low-risk tiers within 72 h, clinicians can allocate monitoring intensity and interventions more judiciously, potentially reducing unnecessary imaging or prolonged observation in lower-risk patients. This strategy is designed to complement—not replace— established severity scales such as Hunt-Hess or early neuroimaging. When used in combination with our static, admission-based nomogram, dynamic calcium monitoring enables a two-tiered approach: early bedside risk estimation at admission, followed by trajectory-informed refinement within the first 3 days. Crucially, because serum calcium is universally tested and electronically captured, its integration into routine workflows and decision-support tools is both feasible and scalable. The adoption of a calcium-guided, tiered decision algorithm holds promise for earlier identification of delayed hydrocephalus, more timely intervention, and improved outcomes by preventing irreversible neurological deterioration (34).

While dynamic calcium monitoring offers valuable real-time risk refinement, our admission-based prediction model adds complementary strength by enabling early risk stratification using only baseline clinical data. Existing tools for predicting delayed hydrocephalus after SAH—such as the Hunt-Hess grade, WFNS score, or early radiographic signs—primarily reflect initial neurological severity, but often lack the granularity and individualized precision required for early intervention planning (35). Moreover, these scales do not fully capture systemic risk factors that may influence long-term outcomes (36). By incorporating readily available admission variables—including serum calcium, Hunt-Hess grade, and major vascular risk factors (hypertension, diabetes, dyslipidemia, smoking, alcohol use)—our model integrates both acute neurological status and chronic physiological vulnerability. This yields a nomogram with excellent predictive performance (AUC = 0.935 for internal validation, AUC = 0.898 for external validation), outperforming conventional scores or single-factor assessments. Importantly, the model’s reliance on routine clinical parameters ensures high feasibility, facilitating real-time application at the bedside—even in settings lacking advanced imaging or serial lab monitoring. When paired with 72-h calcium trajectories, the admission-based model forms a tiered framework for individualized decision-making: early classification on admission supports immediate triage, while adjustment informed by dynamic monitoring during the first week allows escalation or de-escalation of surveillance and intervention. This integrated approach represents a significant step forward from traditional models that depend solely on static neurological assessment or delayed imaging changes, enabling timelier, resource-efficient, and patient-centered management of delayed hydrocephalus risk (18, 37).

The predictive value of serial hypocalcemia was evaluated against potential confounding by nimodipine or hyponatremia. First, nimodipine use was near-universal and thus cannot explain the differential calcium trends. Second, the trajectory of serum calcium decline was not significantly altered by hyponatremia status. This indicates that hypocalcemia itself, rather than being a surrogate for these factors, has an independent association with hydrocephalus risk.

Despite its clinical relevance and strong predictive performance, this study has several limitations that warrant consideration. First, because the primary outcome required 90 ± 14-day follow-up neuroimaging for ascertainment, patients who died before the scheduled follow-up assessment or lacked follow-up neuroimaging were not evaluable for the primary outcome; this may introduce survivorship bias and limit the generalizability of our findings. Second, although our cohort was rigorously predefined with standardized protocols and externally validated, the external validation was limited to two centers, which may constrain generalizability across broader healthcare settings or populations with differing SAH etiologies and care protocols. Future multicenter studies with larger, geographically and ethnically diverse cohorts are warranted. Third, while serum calcium was measured at multiple time points, we did not assess ionized calcium or parathyroid hormone levels, which may more directly reflect calcium bioavailability and endocrine regulation in the acute post-SAH phase (38). Fourth, although we demonstrated strong statistical performance, the study design remains observational; therefore, a causal relationship between hypocalcemia and delayed hydrocephalus cannot be established, and experimental/translational studies are needed to determine whether correcting calcium deficits can modify risk. Lastly, we did not include detailed neuroimaging features (e.g., third ventricular width, blood load scores) or advanced biomarkers (e.g., neuroinflammatory cytokines, CSF flow metrics) that might further enhance model precision. Future efforts should aim to integrate multimodal data—clinical, biochemical, imaging, and molecular—to build more comprehensive and dynamic prediction platforms for delayed hydrocephalus.

## Conclusion

This study identifies early dynamic changes in serum calcium—particularly hypocalcemia at 72 h—as a strong and practical predictor of delayed hydrocephalus following SAH. Both dynamic serum calcium monitoring and a nomogram derived from admission variables demonstrated robust predictive performance and clinical applicability. By integrating these static and dynamic risk markers, clinicians may achieve earlier, more personalized delayed hydrocephalus risk stratification and optimize surveillance and intervention strategies. These findings underscore the potential of serum calcium as an accessible biomarker and modifiable target in post-SAH management, while also highlighting the need for future multicenter validation and mechanistic exploration.

## Data Availability

Publicly available datasets were analyzed in this study. This data can be found here: the datasets generated and/or analyzed during the current study are not publicly available due to patient privacy and institutional data protection policies but are available from the corresponding author on reasonable request. Any request for access to de-identified data will be subject to approval by the Tianjin Medical University General Hospital Ethics Committee and execution of a data use agreement.

## Footnotes

1
https://www.python.org

2
https://www.r-project.org

## Supplementary material

SUPPLEMENTARY FIGURE S1 External validation of the predictive model for delayed hydrocephalus in a pooled external cohort. This figure illustrates the performance of the locked model (no refitting) in a pooled cohort of 142 patients aggregated from two hospitals. **(A)** ROC curve analysis showing good model discrimination with a ROC AUC of 0.898. **(B)** Precision-recall curve analysis with a PR AUC of 0.739, reflecting the model’s performance in the validation cohort. **(C)** Confusion matrix showing the classification metrics: sensitivity, specificity, PPV, and NPV at various threshold probabilities. **(D)** Calibration curve demonstrating strong agreement between predicted and observed values, with a Hosmer-Lemeshow test *p*-value of 0.061, indicating good calibration. **(E)** Decision curve analysis indicating net clinical benefit across clinically relevant threshold probabilities. AUC, area under the curve; DCH, delayed hydrocephalus; NPV, negative predictive value; PPV, positive predictive value; PR, precision-recall; ROC, receiver operating characteristic.

## Glossary

ANOVA: Analysis of variance
AUC: Area under the curve
BBB: Blood–brain barrier
BMI: Body mass index
CI: Confidence interval
CSF: Cerebrospinal fluid
CT: Computed tomography
DCA: Decision curve analysis
DCH: Delayed hydrocephalus
GCS: Glasgow Coma Scale
HL: Hosmer–Lemeshow
IL-6: Interleukin-6
IQR: Interquartile range
LASSO: Least absolute shrinkage and selection operator
MICE: Multivariate imputation by chained equations
MMP-9: Matrix metalloproteinase-9
MRI: Magnetic resonance imaging
Na: Sodium
NPV: Negative predictive value
PPV: Positive predictive value
PR: Precision–recall
ROC: Receiver operating characteristic
SAH: Subarachnoid hemorrhage
SD: Standard deviation
TMUGH: Tianjin Medical University General Hospital
WFNS: World Federation of NeurosurgicalSocieties
