# Supplementary material for: Serial serum calcium dynamics predict delayed hydrocephalus after spontaneous subarachnoid hemorrhage: development and validation of a clinical nomogram in an observational cohort
Source: Front Neurol. 2026 Mar 24;17:1762189. doi: 10.3389/fneur.2026.1762189 (PMC13053312; doi:10.3389/fneur.2026.1762189)
Supplement: Supplementary file 1 [file Table_1.DOCX]

| Table S1: Serum calcium concentration changes at different time points in 302 patients with spontaneous subarachnoid hemorrhage | | | | |
| --- | --- | --- | --- | --- |
| **Variable** | **Admission** | **72 hours** | **1 week** | **P-value** |
| **Serum calcium (mg/dL)** | 9.78 ± 0.95 | 8.92 ± 0.87* | 8.14 ± 0.76^#^ | ＜0.001 |
| *: P < 0.05 vs. Admission; #: P < 0.05 vs. 72 hours | | | | |
