# Supplementary material for: Serial serum calcium dynamics predict delayed hydrocephalus after spontaneous subarachnoid hemorrhage: development and validation of a clinical nomogram in an observational cohort
Source: Front Neurol. 2026 Mar 24;17:1762189. doi: 10.3389/fneur.2026.1762189 (PMC13053312; doi:10.3389/fneur.2026.1762189)
Supplement: Supplementary file 2 [file Table_2.DOCX]

| **Table S2: Longitudinal correlations of serum calcium with neurological severity at different time points in 302 patients with spontaneous subarachnoid hemorrhage.** | | | |
| --- | --- | --- | --- |
| **Timepoint** | **Clinical metric** | **Correlation coefficient (r)** | **P-value** |
| **On admission** | **GCS score** | **0.48** | **0.009** |
|  | **Hunt-Hess grade** | **-0.61** | **0.003** |
| **72 hours** | **GCS score** | **0.65** | **<0.001** |
|  | **Hunt-Hess grade** | **-0.73** | **<0.001** |
| **1 week** | **GCS score** | **0.71** | **<0.001** |
|  | **Hunt-Hess grade** | **-0.79** | **<0.001** |
| **Abbreviations: GCS: Glasgow Coma Scale.** | | | |
