# Supplementary material for: Serial serum calcium dynamics predict delayed hydrocephalus after spontaneous subarachnoid hemorrhage: development and validation of a clinical nomogram in an observational cohort
Source: Front Neurol. 2026 Mar 24;17:1762189. doi: 10.3389/fneur.2026.1762189 (PMC13053312; doi:10.3389/fneur.2026.1762189)
Supplement: Supplementary file 3 [file Table_3.DOCX]

| Table S3: Longitudinal analysis of serum calcium trajectories in spontaneous subarachnoid hemorrhage patients with and without delayed hydrocephalus. | | | | | |
| --- | --- | --- | --- | --- | --- |
| Time Point | Non-Delayed Hydrocephalus  Group  (n=229) | Delayed Hydrocephalus Group  (n=73) | Mean Difference (95% CI) | t-value | P-value |
| Admission (mg/dL) | 9.82 ± 0.98 | 8.15 ± 0.92 | 1.67 (1.42, 1.92) | 13.42 | <0.001 |
| 72 hours (mg/dL) | 10.35 ± 2.11 | 7.28 ± 0.78 | 1.77 (1.56, 1.98) | 16.25 | <0.001 |
| 1 week (mg/dL) | 8.88 ± 1.57 | 7.06 ± 0.71 | 1.62 (1.41, 1.83) | 15.38 | <0.001 |
| P-trend | <0.001 | <0.001 |  |  |  |
| Abbreviations: CI, confidence interval. | | | | | |
