# Supplementary material for: Serial serum calcium dynamics predict delayed hydrocephalus after spontaneous subarachnoid hemorrhage: development and validation of a clinical nomogram in an observational cohort
Source: Front Neurol. 2026 Mar 24;17:1762189. doi: 10.3389/fneur.2026.1762189 (PMC13053312; doi:10.3389/fneur.2026.1762189)
Supplement: Supplementary file 4 [file Table_4.DOCX]

| Table S4: Table S4. Serum calcium quartiles and risk of delayed hydrocephalus after spontaneous subarachnoid hemorrhage: incidence, ROC performance, and diagnostic cut-offs | | | | | | | | | | | | |
| --- | --- | --- | --- | --- | --- | --- | --- | --- | --- | --- | --- | --- |
| Time point | Quartile | Patient profile | | | |  | Diagnostic performance | | | | | |
|  |  | n | Calcium (mg/dL) Mean ± SD | Delayed hydrocephalus cases | Delayed hydrocephalus rate (%) |  | AUC (95% CI) | Optimal Cutoff (mg/dL) | Sensitivity (%) | Specificity (%) | PPV (%) | NPV (%) |
| **Admission** | Q1 (≤8.75) | 76 | 8.15 ± 0.92 | 42 | 55.3 |  | 0.648 (0.568-0.728) | 8.25 | 76.2 | 58.8 | 68.9 | 67.1 |
|  | Q2 (8.76-9.15) | 75 | 8.95 ± 0.35 | 17 | 22.7 |  | 0.623 (0.532-0.714) | 8.90 | 70.6 | 55.2 | 45.7 | 77.6 |
|  | Q3 (9.16-9.85) | 75 | 9.52 ± 0.25 | 8 | 10.7 |  | 0.584 (0.482-0.686) | 9.40 | 62.5 | 53.7 | 28.6 | 82.9 |
|  | Q4 (≥9.86) | 76 | 10.28 ± 0.45 | 3 | 3.9 |  | 0.517 (0.407-0.627) | 10.10 | 66.7 | 49.3 | 14.3 | 92.1 |
| **72 hours** | **Q1 (≤7.65)** | **76** | **7.28 ± 0.78** | **52** | ****68.4**** |  | ****0.853 (0.792-0.914)**** | ****7.35**** | ****84.6**** | ****76.9**** | ****82.5**** | ****79.5**** |
|  | Q2 (7.66-8.10) | 75 | 7.88 ± 0.30 | 32 | 42.7 |  | 0.748 (0.672-0.824) | 7.80 | 78.1 | 67.4 | 65.3 | 79.5 |
|  | Q3 (8.11-8.65) | 76 | 8.38 ± 0.35 | 18 | 23.7 |  | 0.692 (0.602-0.782) | 8.25 | 72.2 | 62.1 | 46.2 | 83.3 |
|  | Q4 (≥8.66) | 75 | 9.05 ± 0.60 | 6 | 8.0 |  | 0.608 (0.508-0.708) | 8.90 | 66.7 | 56.5 | 25.0 | 88.7 |
| **1 week** | Q1 (≤7.45) | 76 | 7.06 ± 0.71 | 47 | 61.8 |  | 0.782 (0.702-0.862) | 7.15 | 80.9 | 72.4 | 76.9 | 76.7 |
|  | Q2 (7.46-7.85) | 75 | 7.65 ± 0.25 | 29 | 38.7 |  | 0.684 (0.592-0.776) | 7.60 | 75.9 | 60.9 | 58.3 | 77.8 |
|  | Q3 (7.86-8.35) | 76 | 8.10 ± 0.30 | 16 | 21.1 |  | 0.631 (0.532-0.730) | 8.05 | 68.8 | 56.7 | 40.0 | 80.6 |
|  | Q4 (≥8.36) | 75 | 8.72 ± 0.45 | 5 | 6.7 |  | 0.573 (0.462-0.684) | 8.60 | 60.0 | 52.9 | 20.0 | 87.5 |
| Abbreviations: AUC, area under the curve; CI, confidence interval; NPV, negative predictive value; PPV, positive predictive value; SD, standard deviation. | | | | | | | | | | | | |
