# Supplementary material for: Serial serum calcium dynamics predict delayed hydrocephalus after spontaneous subarachnoid hemorrhage: development and validation of a clinical nomogram in an observational cohort
Source: Front Neurol. 2026 Mar 24;17:1762189. doi: 10.3389/fneur.2026.1762189 (PMC13053312; doi:10.3389/fneur.2026.1762189)
Supplement: Supplementary file 5 [file Table_5.DOCX]

| Table S5: Comparison of serial serum calcium levels in subarachnoid hemorrhage patients with and without hyponatremia during hospitalization. | | | | | |
| --- | --- | --- | --- | --- | --- |
| Time Point | Hyponatremia Group (Na < 135 mmol/L, n=106) | Non-Hyponatremia Group (Na ≥ 135 mmol/L, n=196) | Mean Difference (95% CI) | t-value | P-value |
| Admission (mg/dL) | 8.92 ± 0.91 | 8.99 ± 0.97 | -0.07 (-0.28, 0.14) | -0.61 | 0.541 |
| 72 hours (mg/dL) | 8.85 ± 0.88 | 8.94 ± 0.90 | -0.09 (-0.30, 0.12) | -0.82 | 0.412 |
| 1 week (mg/dL) | 8.79 ± 0.84 | 8.83 ± 0.89 | -0.04 (-0.24, 0.16) | -0.40 | 0.689 |
| CI, confidence interval. | | | | | |
