# Supplementary material for: Serial serum calcium dynamics predict delayed hydrocephalus after spontaneous subarachnoid hemorrhage: development and validation of a clinical nomogram in an observational cohort
Source: Front Neurol. 2026 Mar 24;17:1762189. doi: 10.3389/fneur.2026.1762189 (PMC13053312; doi:10.3389/fneur.2026.1762189)
Supplement: Supplementary file 6 [file Table_6.DOCX]

| Table S6: Clinical and demographic characteristics of external cohort patients: non-delayed and delayed hydrocephalus groups included in the nomogram model. | | | | | | | | |
| --- | --- | --- | --- | --- | --- | --- | --- | --- |
| Variable | Total  (n=142) | |  | North China University of Science and Technology Affiliated Hospital  (n=78) | |  | Affiliated Hospital of Qinghai University  (n=64) | |
|  | Non-Delayed Hydrocephalus Group  (n=108) | Delayed Hydrocephalus Group  (n=34) |  | Non-Delayed Hydrocephalus Group  (n=59) | Delayed Hydrocephalus Group  (n=19) |  | Non-Delayed Hydrocephalus Group  (n=49) | Delayed Hydrocephalus Group  (n=15) |
| Serum calcium  (admission) | 9.54±  0.96 | 8.52±  1.05 |  | 9.60±0.96 | 8.64±0.99 |  | 9.46±0.97 | 8.37±1.15 |
| Hypertension | 83(76.9) | 26(76.5) |  | 44(74.6) | 16(84.2) |  | 39(79.6) | 10(66.7) |
| Hunt-Hess grade | 2.3±  1.1 | 2.8±  1.3 |  | 2.4±1.1 | 2.5±1.4 |  | 2.2±1.0 | 3.1±1.2 |
| Smoking history | 9(8.3) | 3(8.8) |  | 4(6.8) | 3(15.8) |  | 5(10.2) | 0(0.0) |
| Dyslipidemia | 5(4.6) | 2(5.9) |  | 3(5.1) | 2(10.5) |  | 2(4.1) | 0(0.0) |
| Alcohol use | 50(46.3) | 13(38.2) |  | 30(50.8) | 7(36.8) |  | 20(40.8) | 6(40.0) |
| Diabetes | 18(16.7) | 6(17.6) |  | 9(15.3) | 3(15.8) |  | 9(18.4) | 3(20.0) |
