# Supplementary figures and images for: Serial serum calcium dynamics predict delayed hydrocephalus after spontaneous subarachnoid hemorrhage: development and validation of a clinical nomogram in an observational cohort
Source: Front Neurol. 2026 Mar 24;17:1762189. doi: 10.3389/fneur.2026.1762189 (PMC13053312; doi:10.3389/fneur.2026.1762189)

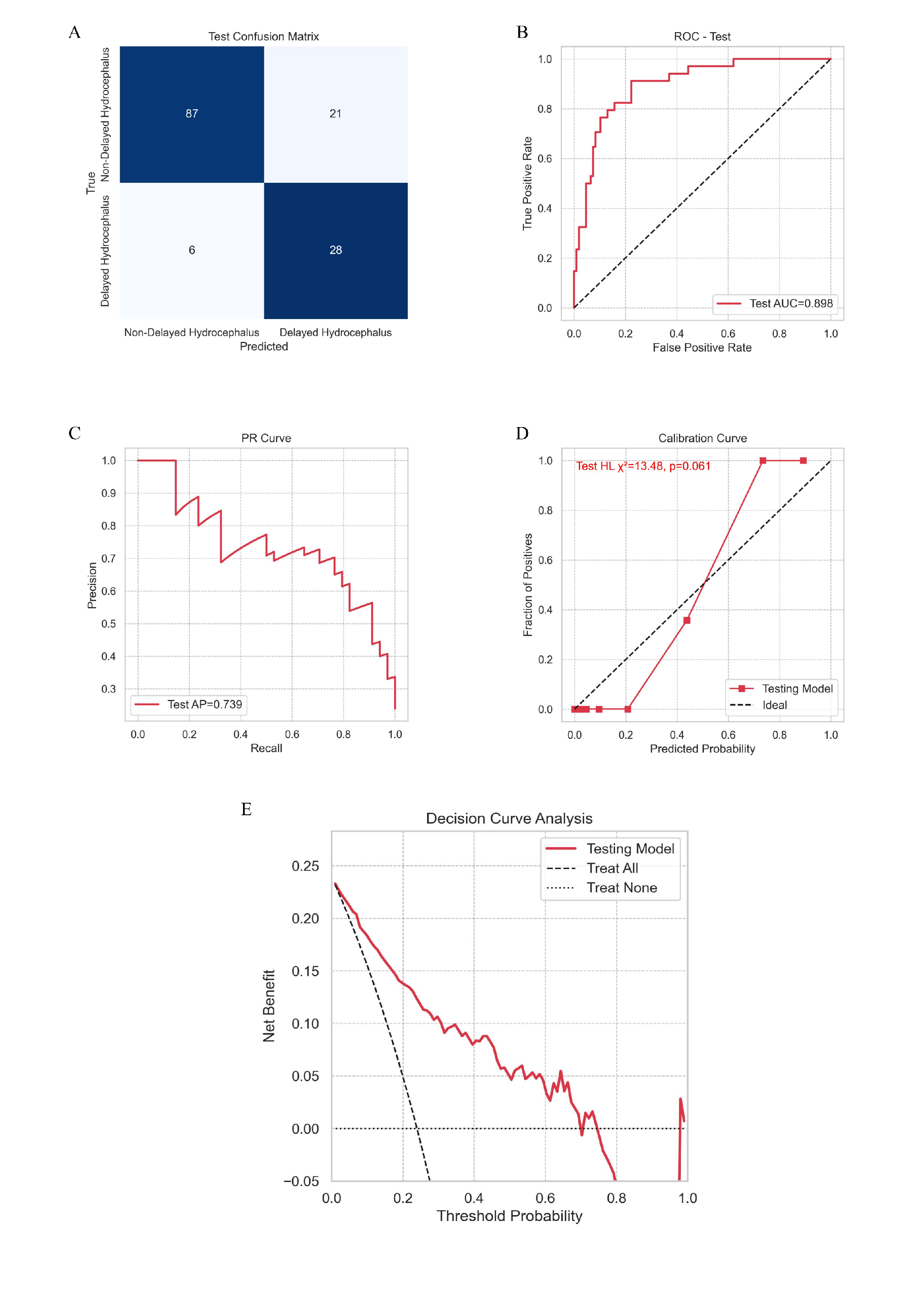

Supplement: SUPPLEMENTARY FIGURE S1 — External validation of the predictive model for delayed hydrocephalus in a pooled external cohort. This figure illustrates the performance of the locked model (no refitting) in a pooled cohort of 142 patients aggregated from two hospitals. (A) ROC curve analysis showing good model discrimination with a ROC AUC of 0.898. (B) Precision-recall curve analysis with a PR AUC of 0.739, reflecting the model’s performance in the validation cohort. (C) Confusion matrix showing the classification metrics: sensitivity, specificity, PPV, and NPV at various threshold probabilities. (D) Calibration curve demonstrating strong agreement between predicted and observed values, with a Hosmer-Lemeshow test p-value of 0.061, indicating good calibration. (E) Decision curve analysis indicating net clinical benefit across clinically relevant threshold probabilities. AUC, area under the curve; DCH, delayed hydrocephalus; NPV, negative predictive value; PPV, positive predictive value; PR, precision-recall; ROC, receiver operating characteristic. [file Image_1.TIFF]
